# Risk factors of emergency cesarean section in pregnant women with severe placenta accreta spectrum: a retrospective cohort study

**DOI:** 10.3389/fmed.2023.1195546

**Published:** 2023-07-05

**Authors:** Hu Zhao, Xin Li, Shuqi Yang, Tianjiao Liu, Jun Zhan, Juan Zou, Changsheng Lin, Yalan Li, Na Du, Xue Xiao

**Affiliations:** ^1^Department of Gynecology and Obstetrics, West China Second University Hospital, Sichuan University, Chengdu, China; ^2^Key Laboratory of Birth Defects and Related Diseases of Women and Children (Sichuan University), Ministry of Education, West China Second Hospital, Sichuan University, Chengdu, China; ^3^Chengdu Women’s and Children’s Central Hospital, School of Medicine, University of Electronic Science and Technology of China, Chengdu, China; ^4^The Fourth People’s Hospital of Chengdu, Psychosomatic Medical Center, Chengdu, China

**Keywords:** placenta accreta spectrum, intraoperative bleeding, emergency cesarean section, pregnancy, activity level

## Abstract

**Introduction:**

Placenta accreta spectrum (PAS) may cause enormous and potentially life-threatening hemorrhage in the intrapartum and postpartum periods in emergency cesarean section. How to reduce the occurrence of emergency cesarean section in patients with severe PAS is the key to reducing the adverse outcomes of them. This study aimed to investigate the impact of emergency cesarean section on the perioperative outcomes of pregnant women with PAS and neonates, and also aimed to explore the risk factors of emergency cesarean section in pregnant women with PAS.

**Materials and methods:**

A retrospective investigation was conducted among 163 pregnant women with severe PAS. Of these, 72 were subjected to emergency cesarean sections. Data on the perioperative characteristics of the mothers and neonates were collected. Multivariable linear regression analysis was used to detect associations between maternal and perioperative characteristics and volume of intraoperative bleeding. Binary logical regression was used to analyze the association between maternal preoperative characteristics and emergency cesarean section. Linear regression analysis is used to analyze the relationship between gestational age and emergency cesarean section.

**Results:**

The risks of emergency cesarean section increase 98, 112, 124, and 62% when the pregnant women with PAS accompanied by GHD, ICP, more prior cesarean deliveries and more severe PAS type, respectively. Noteworthy, the risk of emergency cesarean section decreases 5% when pre-pregnancy BMI increases 1 kg/m^2^ (OR: 0.95; CI: 0.82, 0.98; *p* = 0.038). Moreover, there is no significant linear correlation between emergency cesarean section and gestational age.

**Conclusion:**

GHD, ICP, multiple prior cesarean deliveries and severe PAS type may all increase the risk of emergency cesarean section for pregnant women with PAS, while high pre-pregnancy BMI may be a protective factor due to less activity level. For pregnant women with severe PAS accompanied by these high risk factors, more adequate maternal and fetal monitoring should be carried out in the third trimester to reduce the risk of emergency cesarean section.

## Introduction

Placenta accreta spectrum (PAS) is a condition of abnormal placental invasion encompassing the placenta accreta, increta, and percreta, which is a major cause of severe maternal morbidity and mortality (1). PAS has become more common with the increasing rate of cesarean sections (2–4). The incidence of PAS is approximately 1 in 50 if there has been one cesarean section; and one in six after two previous cesarean sections as a result of the increased use of cesarean delivery (5, 6).

Placenta accreta spectrum is one of the most serious long-term complications of cesarean section, which may result in heavy postpartum hemorrhage. Due to endometrial damage, poor wound healing, and other factors, the villi and placenta are more likely to invade the muscle layer and even the serous layer in PAS patients who undergo cesarean section, resulting in placenta implantation and incomplete separation of the placenta during delivery (7–9). The rapture of the placental marginal sinus can result in severe bleeding during the operation, leading to severe complications such as disseminated intravascular coagulation, hysterectomy, infection, and even death (10–13).

It is a necessary but difficult challenge to reduce the amount of intraoperative bleeding in pregnant women with PAS to ensure their safety and postoperative recovery. Therefore, according to the guidelines for pregnant women with severe PAS (14, 15), it is generally recommended to conduct planned cesarean section with adequate preoperative preparation and evaluation in advance. However, under the current management mode, pregnant women with PAS may still undergo emergency surgery for sudden massive bleeding, which is extremely dangerous and can lead to a high risk of hysterectomy and endanger the lives of pregnant women (16, 17).

Thus, in the present study, we investigated the emergency and planned cesarean section characteristics in pregnant women with PAS. Given the findings reported in our study, we aimed to determine the impact of emergency planned cesarean section on the perioperative outcomes of pregnant women with PAS and neonates, and also aimed to explore the risk factors of emergency cesarean section in pregnant women with PAS.

## Methods

### Study design and participants

The present study was embedded in the Longitudinal Placenta Accreta Spectrum Study (LoPASS), an ongoing pregnancy and birth cohort study conducted in Chengdu, which aims to determine the relative contributions of genes and the environment to the development and prognosis of PAS (18–20). This retrospective sub-cohort study was conducted at West China Second University Hospital and included all women diagnosed with PAS during their third trimester between January 2018 and December 2022 (Chinese Clinical Trial Registration Number: ChiCTR2100052428). During the study period, the total number of deliveries per year ranged from 15,000–20,000. This study was approved by the Ethics Committee of West China Second University Hospital (No. 20180117). All methods were performed in accordance with the relevant guidelines and regulations. Written or oral informed consent was obtained from all the participants. This subgroup study included pregnant women with ultrasound-confirmed PAS in the third trimester. Twins and multiple pregnancies were eliminated from this study because of the high rate of poor outcomes (21). Furthermore, PAS with a placenta score of <10 did not meet the indication for severe placenta accreta spectrum in our hospital; therefore, we eliminated these pregnant women from the final analysis. The detailed placental scores of PAS are shown in Supplementary Table S1 (22). Mothers who had chronic metabolic diseases or were taking immunosuppressants and infants with severe malformations or genetic disorders were excluded from the study.

### Data collection

Maternal sociodemographic data (age, height, weight, education, occupation, parity, mode of conception), lifestyle behaviors before pregnancy (smoking and alcohol use), and pre-existing conditions were collected during the first prenatal examination at 12–14 gestational weeks from medical records. Data on pregnancy complications and maternal and neonatal outcomes, including gestational age, preterm premature rupture of membranes (<37 gestational weeks), neonatal sex, intraoperative bleeding (collecting bag), neonatal umbilical arterial blood gas values and birth weight, were collected from medical records.

### Cesarean delivery indications for PAS

Previous research has shown that in pregnant women with PAS delivery before the expected date of childbirth would reduce the incidence of adverse pregnancy outcomes, as compared to expectant management (23, 24). Therefore, in our hospital, we suggest that pregnant women with PAS and without prenatal bleeding should have cesarean delivery during the 36th-week of pregnancy, and pregnant women with PAS and repeated minor prenatal bleeding should have cesarean delivery during the 34th-week of pregnancy. If sudden and massive vaginal bleeding (>100 ml) occurred before the gestational week of the planned cesarean, or if the pregnant woman felt that the fetal movement had significantly reduced and was accompanied by abnormal antenatal fetal monitoring or umbilical cord blood flow, urgent cesarean delivery would be performed.

### Standardized operation process for pregnant women with PAS

#### Preoperation

(1) Placental MRI, transvaginal and transabdominal combined ultrasound placental scoring, blood preparation, multidisciplinary discussions; (2) The chief surgeon performed bedside ultrasound examination before operation to confirm the fetal position, placental position, the relationship between placenta and fetal presentation, evaluate the position of abdominal wall and uterine incision, and make marks; (3) Pregnant women who chosen balloon occlusion were treated with jugular vein catheterization and radial artery puncture to monitor invasive blood pressure.

#### Intraoperation

(1) For patients whose placenta can be avoided, try to select the uterine incision higher than the upper edge of the placenta. If the placenta is widely located in the anterior wall of the uterus, select the thinnest part of the placenta to enter the uterine cavity, and double incision if necessary; (2) After the fetus was delivered, the interventional physician quickly blocked the abdominal aortic balloon (Optional), the first assistant held the uterus out of the abdominal cavity, held the lower segment of the uterus tightly with both hands, and the second assistant tied the lower segment of the uterus with a catheter; (3) The chief surgeon use oval forceps clamp the incision edge of the uterus, push down the bladder, inject oxytocin into the uterine body, wait for the placenta to be naturally stripped. Blunt separation of the placenta with vascular forceps plus fingers, and the action should be gentle to avoid damaging the blood sinus; According to the bleeding situation, it is carried out successively: ligation of the ascending branch of uterine artery, circular suture of the internal orifice of the cervix/cervical lifting suture, woven suture of the anterior wall of the uterus, breakwater like suture of the posterior wall of the uterus, b-lynch suture, intrauterine balloon filling and closing the uterine incision.

#### Postoperation

All PAS patients with bleeding greater than 2000 ml or hysterectomy were admitted to ICU. ICU doctors and obstetricians jointly manage the patient and transfer to obstetrics after the condition is stable.

### Statistical methods

All statistical analyses were performed using SPSS version 25.0 (IBM Corp., Armonk, NY, USA). The chi-squared or Fisher’s exact tests were used to assess categorical data, which were reported as counts and percentages. The means and standard deviations of continuous variables were calculated using the Student’s *t*-test and least significant difference Student’s *t*-test. Multivariable linear regression analysis was used to detect associations between maternal and perioperative characteristics and volume of intraoperative bleeding. Binary logical regression was used to analyze the association between maternal preoperative characteristics and emergency cesarean section. Linear regression analysis is used to analyze the relationship between gestational age and emergency cesarean section. Covariates were selected according to the different variables in the univariate analysis and factors reported in previous studies that would affect the dependent variable. All tests were two-tailed, and statistical significance was set at *p* < 0.05.

## Results

**Figure 1:**
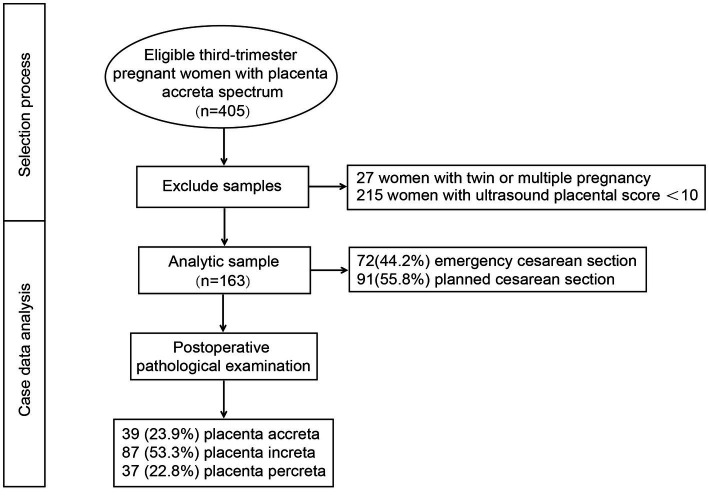
The selection process for this study.

The selection process of the study population is shown in Figure 1. A total of 405 third-trimester pregnant women with PAS from LoPASS were initially recruited into this subgroup study. After excluding mother-offspring pairs that did not match the inclusion criteria and whose ultrasound placental score was <10, the final analysis included 163 mother-offspring pairs. Descriptive data of the study participants are shown in Table 1. The average maternal age at recruitment was 32.48 ± 4.06 years, the average gestational age was 35.89 ± 1.75 weeks, and the average ultrasound placental score was 11.29 ± 1.13. The mean birth weight of all neonates was 2401.90 ± 479.72 g. Furthermore, only 1.9% of mothers conceived *via* assisted reproductive technology, and 87.7% of maternal parity was ≤2 due to the Chinese two-child policy. The average intraoperative bleeding was 1254.04 ± 904.17 ml. Finally, 72 (44.2%) patients who received emergency cesarean section and 91 (55.8%) patients who with planned cesarean section were included in the analysis.

**Table 1:** Description of the maternal and neonatal characteristics.

Variables	Total
Mothers	163
Maternal age (year)	32.48 ± 4.06
Pre-pregnancy BMI (kg/m^2^)	22.57 ± 3.95
Mode of conception	
Natural conception	160(98.1%)
Assisted reproductive technology	3(1.9%)
Gestational weight gain (kg)	13.11 ± 0.66
Gestational age (week)	35.89 ± 1.75
Preterm birth	143(90.2%)
Gravidity	
2	22(13.4%)
3	30(18.4%)
4	55(33.7%)
5+	56(34.5%)
Parity	
1	38(23.3%)
2	105(64.4%)
3+	20(12.3%)
Times of prior cesarean delivery	
1	109(66.9%)
2	51(31.2%)
3+	3(1.9%)
Emergency/planned cesarean section	
Planned cesarean section	91(55.8%)
Emergency cesarean section	72(44.2%)
Mode of anesthesia	
General anesthesia	95(58.3%)
Regional anesthesia	68(41.7%)
Placental score	11.29 ± 1.13
Intraoperative bleeding	1254.04 ± 904.17
Neonatal birthweight (g)	2401.90 ± 479.72

In emergency cesarean section group, over 80% of the women had prenatal bleeding and above 90% of their neonates were born prematurely; this group had more prior cesarean section number, with a lower pre-pregnancy BMI, gestational age and neonatal birthweights. During the perioperative period, the group had more bleeding volume, blood product transfusion rate, uterine artery ligation rate, hysterectomy rate and ICU transfer rate (Tables 2, 3).

**Table 2:** Description of the maternal and neonatal characteristics by operation types.

Variables	Emergency CS	Planned CS	Value of *p*
Mothers	*N* = 72	*N* = 91	
Age (year)	32.43 ± 3.89	32.55 ± 3.82	0.842^a^
Pre-pregnancy BMI (kg/m^2^)	21.83 ± 3.71	23.10 ± 3.65	0.022^a^
Mode of conception			0.966^c^
Natural conception	71(98.6%)	89(97.8%)	
ART	1(1.4%)	2(2.2%)	
Gestational weight gain (kg)	12.96 ± 3.46	13.46 ± 3.61	0.626^a^
Gestational age (week)	35.35 ± 1.58	36.68 ± 1.60	<0.001^a^
Preterm birth	66(91.2%)	77(84.6%)	<0.001^b^
Smoking	5(6.9%)	4(4.4%)	0.873^c^
Pregnancy-induced illness			
GDM	8(11.1%)	10(11.0%)	0.857^b^
GHD	14(19.4%)	6(6.6%)	0.010^b^
ICP	17(23.6%)	9(9.8%)	0.022^b^
FGR	5(6.9%)	4(4.4%)	0.796^c^
Gravidity	3(3)	2(2)	0.214^d^
Parity	2(2)	2(2)	0.230^d^
Number of prior cesarean sections	2(1)	1(1)	0.040^d^
Number of prior D&C abortions	1(1)	1(2)	0.354^d^
Prenatal bleeding rate	60(83.3%)	23(25.3%)	<0.001^b^
Gestational age of first prenatal bleeding	29.71(4.49)	30.54(6.42)	0.281^d^
Mode of delivery (cesarean section)	72(100%)	91(100%)	–
Infants			
Birthweight (g)	2387.43 ± 522.40	2551.19 ± 569.85	<0.001^a^
Birthlength (cm)	46.28 ± 2.61	47.64 ± 2.79	0.068^a^

BMI, body mass index; ART, assisted reproductive technology; GDM, gestational diabetes; GHD, gestational hypertension disorder; ICP, intrahepatic cholestasis of pregnancy; FGR, fetal growth restriction; PP, placenta previa; D&C, dilation & curettage. ^a^Average and standard deviation, Student’s *t*-test. ^b^Number (percentage), chi-squared test. ^c^Number (percentage), fisher exact test. ^d^Median (interquartile range), Kruskal–Wallis test.

**Table 3:** Description of the perioperative characteristics by operation types.

Variables	Emergency CS *N* = 72	Planned CS *N* = 91	Value of *p*
Preoperation			
Placental score	11.21 ± 1.192	11.40 ± 1.095	0.627^a^
Hemoglobin concentration	114.94 ± 13.695	111.59 ± 12.112	0.100^a^
Mode of anesthesia (General anesthesia)	55(76.4%)	13(14.3%)	<0.001^c^
Prophylactic balloon occlusion	10(13.9%)	62(68.1%)	<0.001^c^
Intraoperation			
Incision mode (Longitudinal incision)	13(18.1%)	11(12.1%)	0.625^b^
Bleeding volume	1412.50 ± 1061.614	1061.54 ± 762.419	0.023^a^
Duration of surgery	87.63 ± 47.631	78.33 ± 43.313	0.136^a^
Urine volume	333.68 ± 242.143	296.76 ± 291.171	0.388^a^
Fluid transfusion volume	2738.47 ± 896.019	2539.01 ± 790.598	0.134^a^
Blood product transfusion	41(56.9%)	33(36.3%)	0.039^b^
Intrauterine balloon	46(63.9%)	61(67.0%)	0.675^b^
Uterine artery ligation	21(29.2%)	14(15.4%)	0.043^b^
Caesarean hysterectomy	19(26.4%)	7(7.7%)	0.005^c^
Neonatal asphyxia	16(22.2%)	10(10.1%)	0.096^c^
Apgar score			
1 min	7.94 ± 0.62	8.81 ± 0.58	0.004^a^
5 min	9.04 ± 0.59	9.35 ± 0.51	0.109^a^
10 min	9.17 ± 0.57	9.40 ± 0.55	0.317^a^
Postoperation			
Antibiotic use time	95.45 ± 54.306	78.75 ± 47.372	0.013^a^
Transfer to ICU	24(33.3%)	10(11.0%)	0.029^b^
24-h bleeding volume	1632.79 ± 881.376	1149.53 ± 1073.559	0.447^a^
Febrile morbidity	25(34.7%)	17(18.7%)	0.076^b^
Hemoglobin reduction	21.84 ± 13.956	18.17 ± 15.442	0.382^a^
Death	0(0%)	0(0%)	–

^a^Average and standard deviation, student’s *t*-test. ^b^Number (percentage), chi-squared test. ^c^Number (percentage), fisher exact test.

Significant differences in the prophylactic balloon occlusion rate, mode of anesthesia, and 1-min Apgar score were found between the emergency and planned cesarean section groups (Table 3). Only 13.9% were received prophylactic balloon occlusion in emergency cesarean section group, whereas the rate was 68.1% in the planned cesarean section group. Moreover, there was a significant difference (*p* < 0.001) in the general anesthesia rate between the two groups (76.4% in emergency cesarean section group and 14.3% in planned cesarean section group). Noteworthy, a significant difference (*p* = 0.004) was found in the 1-min Apgar scores between the two groups but there was no significant difference in the 5- or 10-min Apgar scores. Meanwhile, no significant difference was found in neonatal umbilical arterial blood gas values between the two groups.

**Figure 2:**
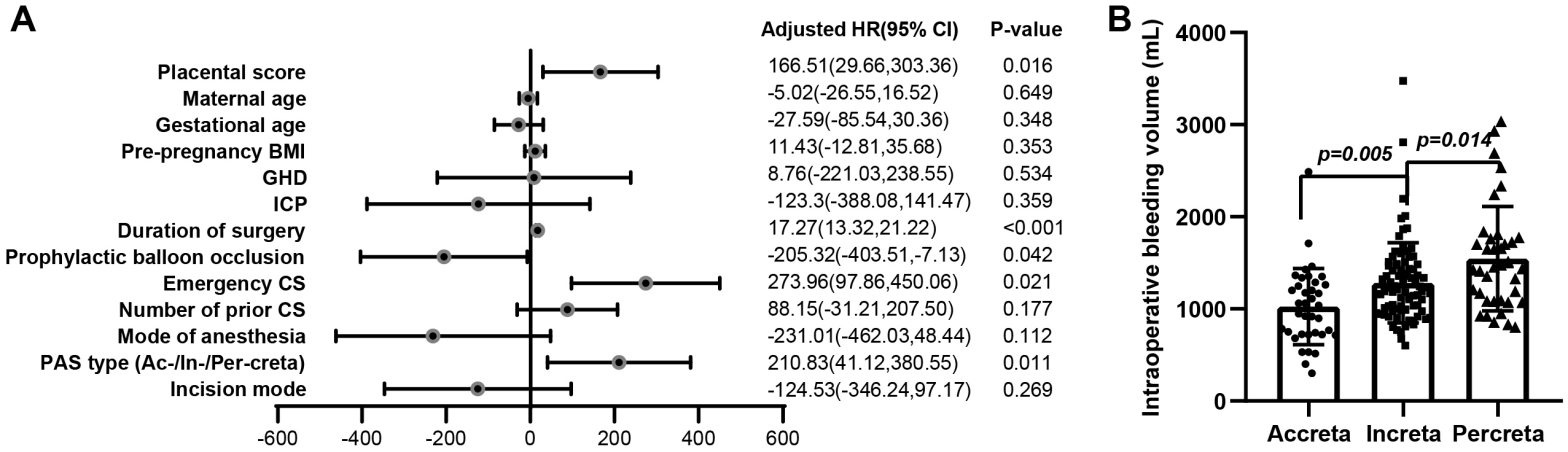
Association between maternal and perioperative characteristics and intraoperative bleeding volume. **(A)** After adjusting for maternal age, pre-pregnancy BMI, gestational age, mode of anesthesia, GHD, ICP, and incision, the results suggested a significant correlation between intraoperative bleeding volume and ultrasound placental score, duration of surgery, preoperative balloon occlusion, emergency cesarean section and PAS type. **(B)** The amount of intraoperative bleeding was significantly different among placenta accreta, increta, and percreta groups (*p* = 0.005 Accreta vs. Increta; *p* = 0.014 Increta vs. Percreta).

In univariate analysis, there were significant differences in intraoperative bleeding between emergency and planned cesarean section group (*p* = 0.023). After adjusting for maternal age, pre-pregnancy BMI, gestational age, mode of anesthesia, GHD, ICP, and incision, the results suggested a significant correlation between intraoperative bleeding volume and ultrasound placental score, duration of surgery, preoperative balloon occlusion, emergency cesarean section and PAS type (Figure 2A). The volume of intraoperative bleeding was positively correlated with the placental score, duration of surgery, emergency cesarean section and PAS type, with the bleeding volume increasing by 167 ml when the placental score increased by 1 point (Beta: 166.51; CI: 29.66, 303.36; *p* = 0.016), and the bleeding volume increased by 17 mL when the duration of surgery increased by 1 min (Beta: 17.27; CI: 13.32, 21.22; *p* < 0.001). The amount of bleeding was significantly reduced by 205 mL in the balloon occlusion group (Beta: –205.32; CI: −403.51, –7.13; *p* = 0.042). Moreover, intraoperative bleeding significantly increases by 274 ml when emergency cesarean section occurs (Beta: 273.96; CI: 97.86, 450.06; *p* = 0.021), and increases by 211 ml when the type of PAS is more severe (Beta: 210.83; CI: 41.12, 380.55; *p* = 0.011; Figure 2B).

Considering that emergency cesarean section in patients with severe PAS may lead to more intraoperative bleeding and hysterectomy rate, the risk factors for emergency cesarean section in patients with severe PAS were further analyzed (Table 4). After adjusting for maternal age, mode of conception, gestational weight gain, number of gravidity, parity and D&C abortions, the results suggested a significant correlation between emergency cesarean section and pre-pregnancy BMI, GHD, ICP, number of prior cesarean deliveries and PAS type. The risks of emergency cesarean section increase 98, 112, 124, and 62% when the pregnant women with GHD, ICP, more prior cesarean deliveries and more severe PAS type, respectively. Noteworthy, the risk of emergency cesarean section decreases 5% when pre-pregnancy BMI increases 1 kg/m^2^ (OR: 0.95; CI: 0.82, 0.98; *p* = 0.038). For pregnant women with PAS, the recommended termination of pregnancy is 36 weeks, and most emergency surgeries were earlier than 36 weeks. Wherefore, the gestational age is an artificial factor and not an independent variable, so it is not included in the binary logistic regression analysis.

**Table 4:** Association between maternal preoperative characteristics and emergency cesarean section.

Variables	Exp(B)	95% CI	*P*-value
Maternal age (year)	1.52	(0.58,2.92)	0.438
Pre-pregnancy BMI (kg/m^2^)	0.95	(0.82,0.98)	0.038
Mode of conception	0.76	(0.47,1.56)	0.261
Gestational weight gain (kg)	1.35	(0.71,1.68)	0.637
GDM	1.10	(0.85,2.60)	0.514
GHD	1.98	(1.13,6.91)	0.038
ICP	2.12	(1.50,7.84)	0.024
Gravidity	0.56	(0.21,1.57)	0.320
Parity	0.91	(0.11,2.34)	0.191
Number of prior cesarean deliveries	2.24	(1.21,7.50)	<0.001
Number of D&C abortions	1.17	(0.40,2.13)	0.794
PAS type (Ac-/In-/Per-creta)	1.62	(1.39,4.71)	0.009

GDM, gestational diabetes; GHD, gestational hypertension disorder; ICP, intrahepatic cholestasis of pregnancy, PAS, placenta accreta spectrum.

**Figure 3:**
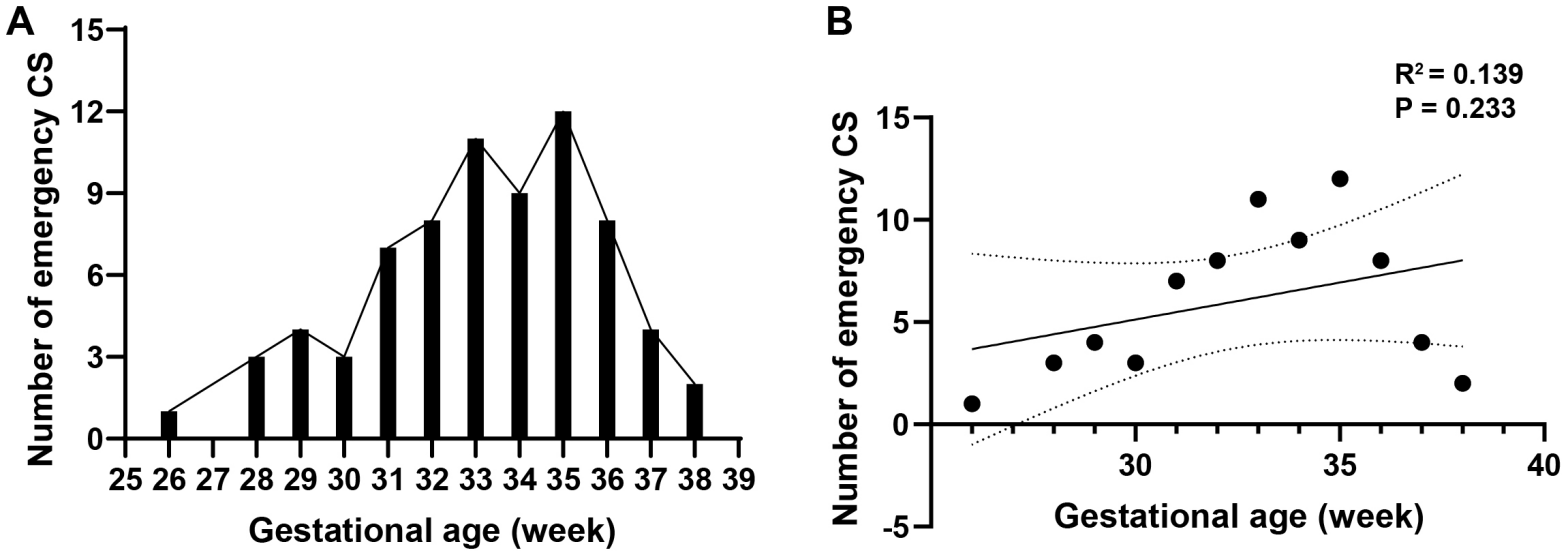
Association between gestational age and emergency cesarean section. **(A)** The gestational age distribution of emergency cesarean section is shown. **(B)** Linear regression analysis is used to analyze the relationship between gestational age and emergency cesarean section, and the results showed that there was no significant correlation between gestational weeks and emergency cesarean section (*R*^2^ = 0.139, *p* = 0.233).

The gestational age distribution of emergency cesarean section is shown in Figure 3A. Linear regression analysis is used to analyze the relationship between gestational age and emergency cesarean section, and the results showed that there was no significant correlation between gestational weeks and emergency cesarean section (*R*^2^ = 0.139, *p* = 0.233; Figure 3B).

## Discussion

In this retrospective preliminary study, we investigated the emergency and planned cesarean section characteristics in pregnant women with PAS. We further analyzed the impact of emergency and planned cesarean section on the perioperative outcomes of pregnant women with PAS and neonates, and also aimed to explore the risk factors of emergency cesarean section in pregnant women with PAS.

Placenta accreta spectrum is a clinical diagnosis and subject to enormous variation based on different diagnostic criteria. The most rigorous classification used to date (ISUOG) considers that PAS cannot be confirmed in the absence of hysterectomy (25, 26). However, in our study, there were only 26 (16.0%) cases of hysterectomy, so we tend to conduct a more detailed preoperative evaluation of patients. In our hospital, the condition of the placenta and uterus was determined by ultrasound and magnetic resonance imaging before surgery, and the placenta was scored through ultrasonographic examination (8, 27–29). The accuracy of ultrasound in preoperative diagnosis of PAS reached 83.3–89.6% (14, 25), and we only included pregnant women with severe PAS (placental score ≥ 10 by ultrasonic), the PAS diagnosis of the patient is relatively reliable.

Pregnant women with a placental score ≥ 10 were considered to be at risk of massive intraoperative bleeding and advised to undergo preoperative balloon occlusion (30, 31). However, during emergency surgery, there may not enough time for interventional physician to conduct prophylactic balloon occlusion, so relatively less choice is made to use balloons. Similar to previous studies (3, 32–34) our study found that prophylactic balloon occlusion can significantly reduce intraoperative bleeding.

Emergency cesarean section in patients with severe PAS may lead to more intraoperative bleeding and hysterectomy rate. In our study, the hysterectomy rate is 7.7% in planned cesarean section while 26.4% in emergency cesarean section, which increases approximately 3.4 times. For planned cesarean section in patients with severe PAS, a multidisciplinary team was formed in our hospital to ensure sufficient supply of blood products, coagulation factors, and equipment, which included hospital staff from the obstetrics, urology, anesthesia, neonatology, and the blood bank departments. Adequate preoperative and blood preparation allows the surgeon a greater opportunity and confidence to retain the uterus, while there are few options left for the surgeon in emergency cesarean section in order to protect the safety of patients. Meanwhile, the amount of intraoperative bleeding in pregnant women with severe PAS at our hospital was much lower than that in previous study (35–37). This can be explained by the greater professional surgical ability of PAS and multidisciplinary cooperation (6). Thus, we suggest that complex surgeries, such as PAS, should be transferred to a more professional hospital to ensure the safety of pregnant women and fetuses and better postoperative recovery.

How to reduce the occurrence of emergency cesarean section in patients with severe PAS is the key to reducing the adverse outcomes of them. In this study, GHD, ICP, multiple prior cesarean deliveries and severe PAS may all increase the risk of emergency cesarean section for pregnant women with PAS, which is similar to previous studies (38–40). Due to the pathological mechanisms of GHD and ICP, them may induce fetal movement decrease, abnormal antenatal fetal monitoring or umbilical cord blood flow. In these cases, emergency cesarean section may occur. Pregnant women only suffer from GHD or ICP, and it is usually recommended to actively terminate pregnancy after 37 and 38 weeks of pregnancy, respectively. However, our findings suggest that PAS pregnant women with GHD or ICP may increase their risk of emergency cesarean section. This means that for this patients, earlier planned cesarean section, such as 34 weeks of pregnancy, may be considered. Moreover, more adequate maternal and fetal monitoring should be carried out in the third trimester to reduce the risk of emergency cesarean section.

However, high pre-pregnancy BMI is a protective factor for emergency cesarean section, which may due to relatively less activity among women with higher pre-pregnancy BMI in the third trimester of pregnancy (41, 42). Nevertheless, since this study was a retrospective study, data of the activity level in the third trimester of pregnancy was not collected, we could not determine the impact of the activity level on emergency cesarean section in pregnant women with PAS. Further prospective cohort studies can be used to clarify their relationship.

Meanwhile, there is no significant linear correlation between emergency cesarean section and gestational age, so before 36 weeks of gestation, it is recommended to extend gestational age as much as possible when the pregnant woman’s condition is stable to reduce adverse fetal outcomes. Moreover, our study population includes PAS patients who underwent surgery at more than 36 weeks of pregnancy. In fact, our hospital does suggest that PAS pregnant women terminate their pregnancy by cesarean section at 34^+0^–35^+6^ weeks of gestation according to the AJOG guidelines. However, due to the huge differences in medical resources among regions in China, some regions (such as Sichuan Aba Tibetan Autonomous Prefecture) have no ability to perform severe PAS cesarean section, and even some pregnant women with PAS have not undergone prenatal examination. These pregnant women were transferred to our hospital for cesarean section in the third trimester of pregnancy, so our study population includes patients who underwent surgery at more than 36 weeks of pregnancy.

The merits of our study are the specialized study population and the large standard sample size. The participants were screened using strict inclusion and exclusion criteria for this retrospective study. Women pregnant with twins were not selected due to a higher risk of maternal and fetal complications. All cesarean sections were performed according to the same surgical procedure, remaining relative consistency of intraoperative information. Additionally, comprehensive maternal and perioperative data were analyzed, resulting in a relatively complete study design. Due to the low natural incidence of PAS, obtaining participants is time-consuming. As a result, it is difficult to recruit and follow-up with such a large sample of pregnant women with PAS at a single hospital for 4 years. The advantage to our study was that we were able to examine such important diseases using large samples due to our hospital being the largest and most professional women’s and children’s hospital in Southwest China. We treat a large number of pregnant women with serious complications. The total number of deliveries annually in our hospital during the study period ranged from 15,000 to 20,000.

This preliminary study adds to our understanding of the risk factors of emergency cesarean section in pregnant women with severe PAS; however, it has some limitations that should be considered. First, the sample size in this study was relatively modest compared with similar studies on common diseases of pregnancy. Second, due to the fact that pregnant women with PAS are recommended to have a planned cesarean section at 36 weeks, data for larger gestational weeks are relatively limited. Third, this study is a retrospective study. Prospective follow-up of pregnant women with severe PAS can more clearly clarify the risk factors of emergency cesarean section in pregnant women with PAS, a large-scale study involving more patients and different types of PAS conducted in multiple centers is required.

## Conclusion

This study suggested that GHD, ICP, multiple prior cesarean deliveries and severe PAS type may all increase the risk of emergency cesarean section for pregnant women with PAS. Meanwhile, there is no significant linear correlation between emergency cesarean section and gestational age, so before 36 weeks of gestation, it is recommended to extend gestational age as much as possible when the pregnant woman’s condition is stable to reduce adverse fetal outcomes. For pregnant women with severe PAS accompanied by these high risk factors, more adequate maternal and fetal monitoring should be carried out in the third trimester to reduce the risk of emergency cesarean section.

## Data availability statement

The original contributions presented in the study are included in the article/Supplementary material, further inquiries can be directed to the corresponding author.

## Author contributions

XX made the data collection. HZ, XL, and SY contributed to statistical analysis. All the authors have participated to study design and manuscript writing and editing, and approved the final version of the manuscript.

## Funding

Financial support for this work was provided by National Natural Science Foundation of China (82071651), National Key Research and Development Program (2022YFC3600304 and 2022YFC2704700), Sichuan Provincial Department of Science and Technology (2023YFS0219 and 2023YFS0228), and Chengdu Science and Technology Bureau (2017-GH02-00030-HZ). The funding agencies did not have any role in the design of the study, the collection, analysis, or interpretation of data, or in writing the manuscript.

## Conflict of interest

The authors declare that the research was conducted in the absence of any commercial or financial relationships that could be construed as a potential conflict of interest.

## Publisher’s note

All claims expressed in this article are solely those of the authors and do not necessarily represent those of their affiliated organizations, or those of the publisher, the editors and the reviewers. Any product that may be evaluated in this article, or claim that may be made by its manufacturer, is not guaranteed or endorsed by the publisher.

## Abbreviations

PAS: placenta accreta spectrum
LoPASS: longitudinal placenta accreta spectrum study
OR: odds ratio
CI: confidence interval
BMI: body mass index
ART: assisted reproductive technology
GDM: gestational diabetes mellitus
GHD: gestational hypertension disorder
ICP: intrahepatic cholestasis of pregnancy
FGR: fetal growth restriction
D&C: dilation & curettage
