# Supplementary material for: Risk factors of emergency cesarean section in pregnant women with severe placenta accreta spectrum: a retrospective cohort study
Source: Front Med (Lausanne). 2023 Jul 5;10:1195546. doi: 10.3389/fmed.2023.1195546 (PMC10370267; doi:10.3389/fmed.2023.1195546)
Supplement: Supplementary file 1 [file Data_Sheet_1.docx]

**Supplementary table 1 Description of the** **placental scores of** **placenta accreta spectrum by** **ultrasound**

| Variables | 0 score | 1 score | 2 score |
| --- | --- | --- | --- |
| Placental relationship with internal cervical os | ≥ 2 cm | Low-lying | Previa |
| Myometrial thinning | Myometrium > 1 mm | Myometrium < 1 mm | Absent |
| Focal exophytic mass and/or placental bulge | Absent | - | Present |
| Utero-vescical hypervascularity | Absent | Increased | Multidirectional flow with bridging vessels |
| Placental lacunae | Not seen | 2–3, regular ≤2 cm | 4–6, irregular, 4 cm |
| Hypoechoic retroplacental space ("clear zone") | Present | Irregular | Absent |
| Subplacental hypervascularity | Normal | Increased with numerous vases, tortuous | Bridging vessels with perpendicular course |
| Hyperechoic uterus–bladder interface (bladder line) | Line clear and complete | Line vague or irregular | Line lost |
| Diffuse or focal turbulent flow in the lacunae | Absent | Focal turbulent flow | Diffuse turbulent flow with feeding vessels |
| Previous cesarean section | - | 1 times | ≥ 2 times |
| Total |  |  |  |
